# Long non-coding RNA TPT1-AS1 promotes angiogenesis and metastasis of colorectal cancer through TPT1-AS1/NF90/VEGFA signaling pathway

**DOI:** 10.18632/aging.103016

**Published:** 2020-04-04

**Authors:** Yiyun Zhang, Jiangyun Sun, Yuan Qi, Yimin Wang, Yu Ding, Kun Wang, Qingxin Zhou, Jingxuan Wang, Fei Ma, Jianguo Zhang, Baoliang Guo

**Affiliations:** 1 Department of Endoscopy, Harbin Medical University Cancer Hospital, Harbin, China; 2 Department of Acupuncture, The First Affiliated Hospital of Harbin Medical University, Harbin, China; 3 Department of General Surgery, The Second Affiliated Hospital of Harbin Medical University, Harbin, China; 4 Department of Central Sterile Supply, The First Affiliated Hospital of Harbin Medical University, Harbin, China; 5 Department of Gastrointestinal Oncology, Harbin Medical University Cancer Hospital, Harbin, China; 6 Department of Medical Oncology, Harbin Medical University Cancer Hospital, Harbin, China

**Keywords:** LncRNA TPT1-AS1, colorectal cancer, NF90, VEGFA, angiogenesis

## Abstract

LncRNAs have been proven closely correlated to tumor progression. A recent study identified LncRNA TPT1-AS1 (TPT1-AS1) as one of the liver-metastasis associated LncRNAs in colorectal cancer (CRC). In this study, we report that TPT1-AS1 is upregulated in CRC tissues, which is associated with poor prognosis. Functional assays unravel a pro-angiogenesis and metastasis role of TPT1-AS1. Mechanistically, Flexmap 3D assays reveal that TPT1-AS1 upregulates the VEGFA secretion in CRC cells. RNA immunoprecipitation and mRNA stability assays further show that TPT1-AS1 interacts with nuclear factor 90 (NF90) and subsequently promotes the association between NF90 and VEGFA mRNA, which leads to the upregulation of VEGFA mRNA stability. Therefore, we elucidate a new regulatory mechanism of TPT1-AS1 in CRC angiogenesis and targeting the TPT1-AS1/NF90/VEGFA axis may provide a useful strategy for diagnosis and treatment for colorectal cancer patients.

## INTRODUCTION

Colorectal cancer (CRC) is the second most common cause of cancer worldwide and lack of detection for early metastasis [1]. Although most CRC patients with early stage are supposed to be cured due to advanced strategies in chemotherapy and target treatment, liver metastasis is common, which essentially dictates the prognosis of CRC patients [2, 3]. Thus, exploring the precise molecular mechanisms that drives CRC metastasis and identifying new therapeutic targets for CRC metastasis are imperative.

It has been revealed that not more than 2% of the human genome are protein coding genes, while the majority of transcripts are non-protein coding RNAs [4]. These transcripts are processed into microRNAs, pseudogenes, or long non-coding RNAs. Increasing evidences indicate that lncRNAs play crucial roles in many cancer cellular processes, such as proliferation, immune response, metastasis, angiogenesis [5, 6]. Recently, a study analyzed the RNA sequencing and microarray data from TCGA database to find differentially expressed lncRNAs in CRC. They identified the liver metastasis-associated lncRNAs, among which Lnc-TPT1-AS1 was significantly upregulated in CRC liver metastasis samples [7]. Thus, we speculated that TPT1-AS1 might play a role in CRC progression. At presents, no researchers have detected the function or regulatory mechanism of TPT1-AS1 in colorectal cancer.

The vascular endothelial growth factor A (VEGFA) belongs to PDGF/VEGF family. It encodes a heparin-binding protein which induces proliferation and migration of vascular endothelial cells. It therefore plays crucial roles in cancer angiogenesis and metastasis [8]. VEGFA expression is known to be regulated through both transcriptional and posttranscriptional mechanisms. The transcriptional activation of VEGFA by HIF-1α under hypoxia condition is well established [9]. However, the mechanisms that the VEGFA mRNA stability is regulated, especially after exporting into the cytoplasm, remain to be studied.

Here, we show that TPT1-AS1 is upregulated in CRC tissues and the enhanced expression of TPT1-AS1 is associated with poor prognosis. Functional studies indicate that TPT1-AS1 promotes CRC angiogenesis and metastasis. Mechanistically, TPT1-AS1 functions as a positive regulator of VEGFA by binding to NF90 and promotes the interaction between NF90 and VEGFA mRNA. These findings might provide new therapeutic strategies for the treatment of CRC.

## RESULTS

### The expression of TPT1-AS1 is upregulated in CRC, which is correlated with poor prognosis

**Figure 1:**
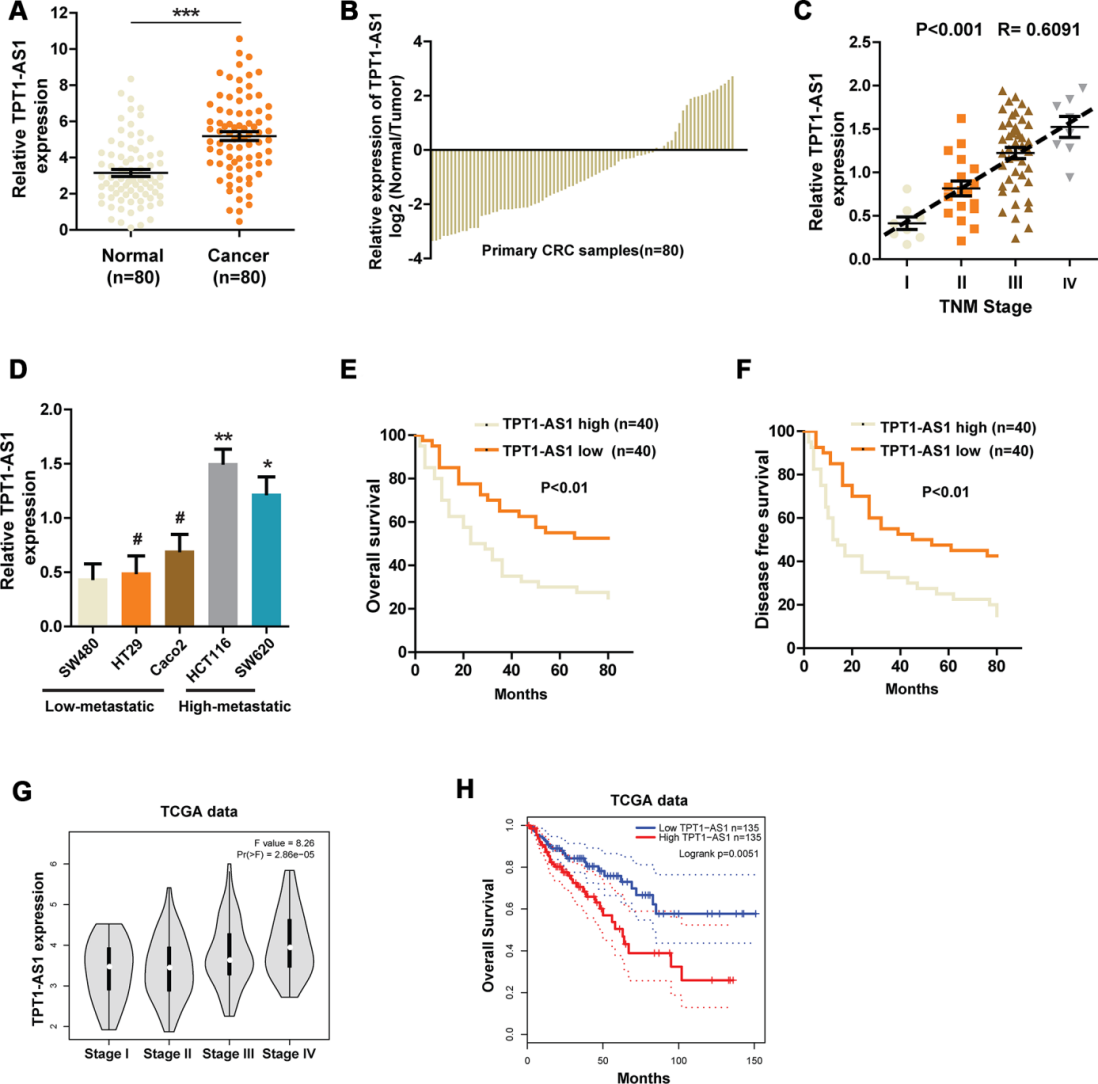
**The expression of TPT1-AS1 in CRC and the relationship between TPT1-AS1 expression and prognosis.** (**A**) and (**B**) qRT- PCR was used to detect the expression of TPT1-AS1 in CRC tissues and corresponding normal tissues. (**C**) Relative TPT1-AS1 expression was examined in CRC tissues from patients of different stages. (**D**) TPT1-AS1 expression was measured in different CRC cell lines using qRT-PCR. Kaplan-Meier plotter was used to analyze the overall survival (**E**) and disease-free survival (**F**) of patients in TPT1-AS1 high/low groups. (**G**) TPT1-AS1 expression pattern in patients of different stages in TCGA database. (**H**) TCGA data was analyzed to obtain the overall survival of patients from TPT1-AS1 high or low groups. *P < 0.05, **P < 0.01, ***P < 0.001, # represents no statistical significance.

Using bioinformatics analysis, TPT1-AS1 was identified as one of the liver metastasis-associated lncRNAs in CRC [7]. However, no experiments have been performed to examine its expression pattern or function in CRC. Thus, we firstly explored the expression of TPT1-AS1 in CRC and corresponding normal tissues in a group of 80 patients. As shown in Figure 1A, qRT-PCR assays uncovered a drastic increase of TPT1-AS1 expression in CRC tissues compared with corresponding normal tissues. Meanwhile, compared with counterparts, the level of TPT1-AS1 was markedly upregulated in 61 of the 80 (76.25%) CRC tissues (Figure 1B). Next, we correlated the TPT1-AS1 expression with clinicopathologic features of the patients. We observed that TPT1-AS1 expression was positively correlated with TNM stage, tumor size, lymph nodes metastasis and distant metastasis (Figure 1C and Table 1). Similarly, we found that the expression of TPT1-AS1 was significantly higher in highly metastatic CRC cell lines than that in low-metastatic cell lines (Figure 1D). Kaplan–Meier analysis was also used to determine the association between TPT1-AS1 expression and patient survival. The results indicated that higher levels of TPT1-AS1 expression were significantly correlated to worse overall survival (OS) and disease-free survival (DFS) (Figure 1E and 1F). Furthermore, we analyzed the data from TCGA dataset to confirm the expression pattern of TPT1-AS1 and its clinical significance in CRC. As expected, the TPT1-AS1 expression was positively associated with TNM stage and the enhanced TPT1-AS1 level predicted a worse prognosis (Figure 1G and 1H). Collectively, these findings suggest that the upregulated TPT1-AS1 might play a crucial role in CRC progression.

**Table 1:** Relationship between TPT1-AS1 expression and clinicopathologic features of CRC patients (n=80).

**Variable**	**Relative TPT1-AS1 expression**		**P-value**
	**Low (n=40)**	**High (n=40)**	
Age			NS
<50	25	19	
>50	15	21	
Gender			NS
Male	22	26	
Female	18	14	
Histological differentiation			NS
Well	16	12	
Moderate	13	19	
Poor	11	9	
Primary tumor sites			NS
Ascending colon	11	7	
Transverse colon	7	5	
Descending colon	3	6	
Sigmoid colon	8	9	
Rectum	11	13	
Tumor size			P<0.05
<5cm	24	13	
>5cm	16	27	
Lymph node metastasis			P<0.01
Yes	17	29	
No	23	11	
Distant metastasis			P<0.01
Yes	14	28	
No	26	12	
Tumor stage			P<0.01
I-II	19	8	
III-IV	21	32	

Note: CRC patients were divided into TPT1-AS1 high group and low group according to the analysis of qRT-PCR detection. NS, not significant between different groups. Differences among variables were evaluated by χ^2^ or Fisher’s exact χ^2^-test.

### TPT1-AS1 promotes the invasion and metastasis of CRC

**Figure 2:**
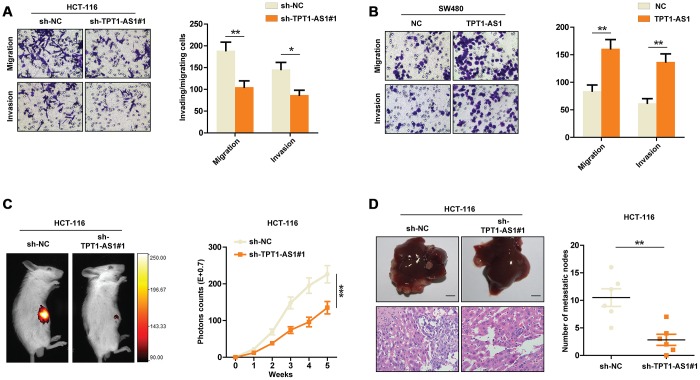
**TPT1-AS1 promotes CRC cells invasion and metastasis.** Transwell assays were performed to detect the migrative and invasive ability of sh-control or TPT1-AS1-knockdown HCT116 cells (**A**) and control or TPT1-AS1-overexpressed SW480 cells (**B**). (**C**) Liver metastasis mice models were used to detect the metastatic ability of HCT116 cells transfected with sh-NC or sh-TPT1-AS1. (**D**) The liver metastatic nodes of indicated mice were counted and HE staining was performed to confirm the tissue type. *P < 0.05, **P < 0.01, ***P < 0.001.

Having observed the relationship between TPT1-AS1 expression and CRC prognosis, we set out to functionally characterize the effects of TPT1-AS1 in CRC. Based on the expression pattern of TPT1-AS1 in the CRC cell lines, we chose SW480 and HCT116 cells for the following functional studies. TPT1-AS1 was stably overexpressed in SW480 cells and silenced by shRNAs in HCT116 cells. The efficiency of overexpression or knockdown in CRC cells was confirmed by real-time PCR assays (Supplementary Figure 1A and 1B). Given that TPT1-AS1 might correlate to liver metastasis in colorectal cancer by genome-wide analysis [7], we detected the potential function of TPT1-AS1 in vitro assays. As indicated in Figure 2A, TPT1-AS1 silencing markedly decreased the migration and invasion of HCT116 cells. In contrast, TPT1-AS1 overexpression significantly enhanced the migrative and invasive ability of SW480 cells in vitro (Figure 2B). Furthermore, we established a liver metastasis mouse model to assess the role of TPT1-AS1 in CRC metastasis in vivo. As expected, the mice injected with sh-TPT1-AS1-transfeted HCT116 cells had less liver metastatic nodes compared with those injected with control cells (Figure 2C and 2D). To conclude, these results indicated that TPT1-AS1 promotes the invasion and metastasis of CRC in vitro and in vivo.

### Knockdown of TPT1-AS1 inhibits the VEGFA expression by decreasing VEGFA mRNA stability

**Figure 3:**
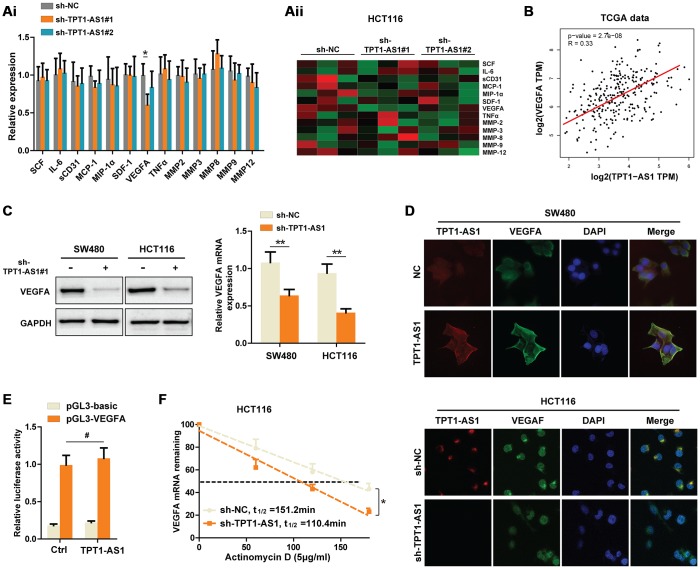
**Knockdown of TPT1-AS1 inhibits the VEGFA expression by decreasing VEGFA mRNA stability.** (**Ai**) Flexmap liquichip assays were performed to detect the metastasis-associated factors in control or sh-TPT1-AS1 HCT116 cell supernatant. The results were also shown in heatmap (**Aii**). (**B**) TCGA date was collected to analyze the relationship between TPT1-AS1 and VEGFA expression. (**C**) Western blot assays and qRT-PCR were used to test the VEGFA expression in indicated CRC cells. (**D**) Combined IF/FISH assays showed that TPT1-AS1 was mainly expressed in cytoplasm of CRC cells and TPT1-AS1 expression positively correlated to VEGFA expression. (**E**) Luciferase reporter assays were used to detect the possibility that TPT1-AS1 transcriptionally regulated VEGFA expression. (**F**) VEGFA mRNA levels were detected using actinomycin D mRNA stability assays in indicated CRC cells. *P < 0.05, **P < 0.01, # represents no statistical significance.

To elucidate the potential mechanisms that TPT1-AS1 promoted CRC metastasis, we detected several well-known factors closely involved in CRC metastasis using Flexmap liquichip assay. As shown in Figure 3Ai and aii, we found that silencing TPT1-AS1 in HCT116 cells markedly decreased the VEGFA secretion in supernatant, but not other factors. A significant positive relationship between TPT1-AS1 and VEGFA was also observed in TCGA database (Figure 3B). Therefore, we next detected the regulatory effect of TPT1-AS1 on VEGFA in CRC cell lines. Interestingly, knockdown of TPT1-AS1 dramatically decreased the protein and mRNA levels of VEGFA in CRC cells (Figure 3C). Given that the subcellular location of long non-coding RNAs generally determines the functions, we used fluorescence in situ hybridization (FISH) of TPT1-AS1 followed by immunofluorescence staining of VEGFA to investigate the mechanisms that TPT1-AS1 regulated VEGFA expression. The results showed that TPT1-AS1 a mainly located in the cytoplasm of CRC cells, where the post-transcriptional regulation of VEGFA is permitted. In addition, overexpression of TPT1-AS1 in SW480 cells markedly upregulated VEGFA expression and vice versa in HCT116 cells (Figure 3D). These results further confirmed the promotional effect of TPT1-AS1 on VEGFA. Again, we performed luciferase reporter assays to exclude the possibility that TPT1-AS1 transcriptionally regulated VEGFA. Not surprisingly, TPT1-AS1 failed to enhance the luciferase activity of pGL-VEGFA-transfected HCT116 cells (Figure 3E). Next, we investigated whether TPT1-AS1 influenced the synthesis or degradation of VEGFA mRNA in CRC cells. Notably, after treated with actinomycin D (an inhibitor of RNA synthesis), TPT1-AS1-silenced HCT116 cells showed a significantly shorter half-life of VEGFA mRNA (Figure 3F). Taken together, our data demonstrated that knockdown of TPT1-AS1 inhibited the VEGFA expression by decreasing VEGFA mRNA stability.

### TPT1-AS1 promotes CRC angiogenesis by upregulating VEGFA expression

**Figure 4:**
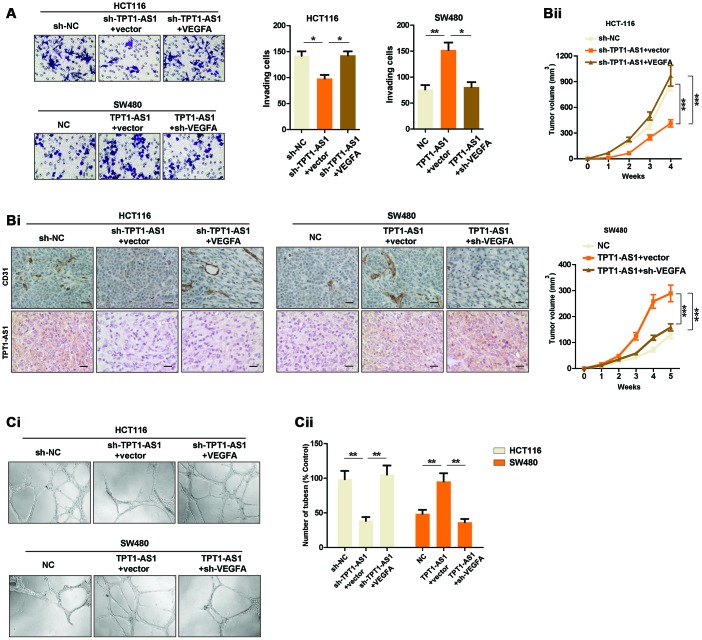
**TPT1-AS1 promotes CRC angiogenesis by upregulating VEGFA expression.** (**A**) Invasion assays was used to show the invasive ability of indicated cells. (**Bi**) IHC and ISH staining were performed to detect CD31 and TPT1-AS1 expression in indicated xenograft tumor tissues. (**Bii**) Subcutaneous tumor models were used to detect the growth rate of indicated CRC cells. (**Ci**) Endothelial tube formation assays were performed using HUVECs to detect the effect of CM from indicated cells. (**Cii**) The number of tubes of each field was counted in indicated groups.

Given that TPT1-AS1 positively regulated VEGFA expression, we set to detect whether TPT1-AS1 affected the angiogenesis and invasion by regulating VEGFA. Transwell assays were used to determine the invasive ability of indicated cells. The results showed that reintroduction of VEGFA in TPT1-AS1-knockdown HCT116 cells restored the number of invading cells, whereas VEGFA depletion significantly reversed the increase of invasive ability of TPT1-AS1-overexpressed SW480 cells (Figure 4A). In addition, the angiogenesis of indicated cells was explored using xenograft mice model. We observed that VEGFA overexpression in TPT1-AS1-silenced HCT116 cells markedly reversed the decreased the subcutaneous tumor growth and CD31 expression in tumors, and vice versa in SW480 cells (Figure 4Bi and 4Bii). The effect of TPT1-AS1 on CRC angiogenesis was further confirmed by endothelial cell tube formation assays. As shown in Figure 4Ci and 4Cii, the condition media (CM) from TPT1-AS1-silenced HCT116 cells dramatically inhibited the tube formation of HUVECs, which was significantly reversed by VEGFA reintroduction in the aforementioned cells. On the contrary, CM from the TPT1-AS1 overexpression groups markedly promoted the tube formation of HUVECs, whereas knockdown of VEGFA in TPT1-AS1-overexpressed SW480 cells revoked the tube formation of indicated cells. In summation, our data indicated that TPT1-AS1 promoted CRC angiogenesis by upregulating VEGFA expression.

### TPT1-AS1 directly interacts with NF90, which enhances the stability of VEGFA mRNA

**Figure 5:**
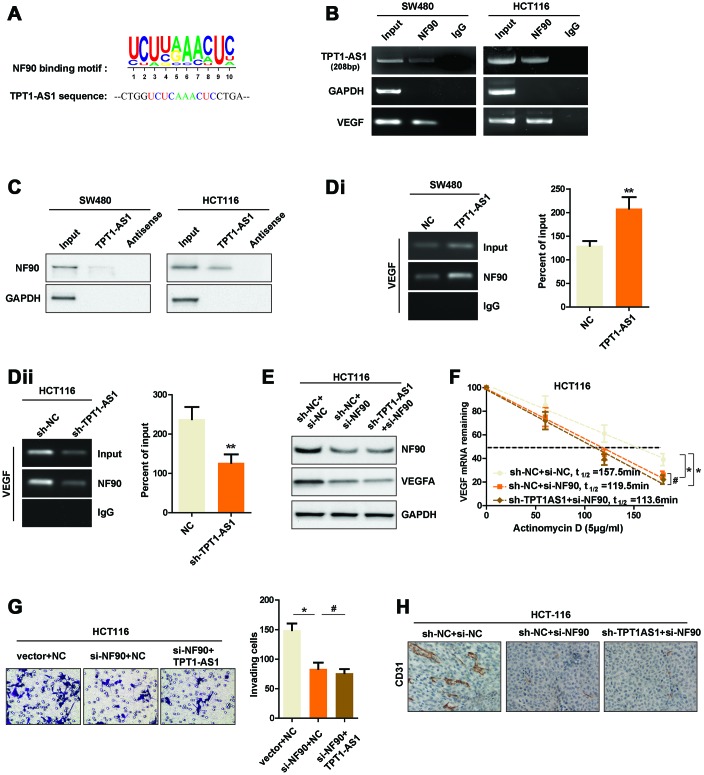
**TPT1-AS1 directly interacts with NF90, which enhances the stability of VEGFA mRNA.** (**A**) The potential binding site that was similar to NF90 conserved binding motif in TPT1-AS1 sequence. (**B**) RIP assays were performed using NF90 antibody or IgG, specific primers were used to detect TPT1-AS1, GAPDH and VEGFA in HCT116 cells. (**C**) RNA pull-down assays were performed using TPT1-AS1 probe or antisense RNA. GAPDH was used as the control. (**Di**) and (**Dii**) RIP assays were performed using anti-NF90 or non-specific IgG in TPT1-AS1 or sh-TPT1-AS1 transfected CRC cells. The RIP-derived VEGFA mRNA was detected using qRT-PCR and presented as a percentage of the input. (**E**) The expression of NF90 and VEGFA were confirmed using western blot assays. (**F**) The half-life of VEGFA mRNA in indicated CRC cells were determined by qRT-PCR. (**G**) Transwell invasion assays were used to determine the invasive ability of indicated cells. (**H**) The CD31 expression of indicated xenograft tumors were detected by IHC staining. **P < 0.01, # represents no statistical significance.

Previous studies reported that NF90, a RNA-binding protein, could bind to VEGFA mRNA, which upregulated the stability of VEGFA mRNA [10]. Our aforementioned data showed that TPT1-AS1 mainly located in the cytoplasm of CRC cells where the post-transcriptional regulation of VEGFA mRNA was permitted. Meanwhile, we also found a potential binding site which was similar to the NF90 conserved binding motif in TPT1-AS1 sequence (Figure 5A). Thus, we speculated that TPT1-AS1 upregulated VEGFA mRNA stability through NF90/VEGFA signaling. To this end, we performed RNA immunoprecipitation assays (RIP) to pull down the NF90-conjuncted nucleotides. We observed a significant enrichment of TPT1-AS1 pulled down by NF90 antibody compared with IgG (Figure 5B). Furthermore, RNA pull-down was performed to confirm the interaction between TPT1-AS1 and NF90. As indicated in Figure 5C, NF90 protein was also pulled down by TPT1-AS1, but not the antisense RNA. To further explore whether TPT1-AS1 affected the interaction between NF90 and VEGFA mRNA, we carried out RIP assays using NF90 antibody in CRC cell lines. As shown in Figure 5Di and 5Dii, overexpression of TPT1-AS1 in SW480 cells significantly upregulated the levels of VEGFA mRNA that was associated with NF90 protein, whereas knockdown of TPT1-AS1 in HCT116 cells decreased the NF90-binding VEGFA mRNA. To test whether TPT1-AS1 regulated the stability of VEGFA mRNA through binding NF90, we detected the half-life of VEGFA mRNA in NF90-silenced HCT116 cells. Before that, we detected the NF90 knockdown efficiency in indicated cells using western blots assays (Figure 5E). Real-time PCR results indicated that knockdown of TPT1-AS1 was unable to decrease the stability of VEGFA mRNA after NF90 knockdown, nor affect the VEGFA protein level (Figure 5E and 5F). Functionally, sh-TPT1-AS1 did not change the angiogenesis or invasive ability of CRC cells (Figure 5G and 5H). In conclusion, the results revealed that TPT1-AS1 interacted with NF90 protein and upregulated the stability of VEGFA mRNA.

### Clinical relations between TPT1-AS1 and VEGFA in CRC patients’ tissues

**Figure 6:**
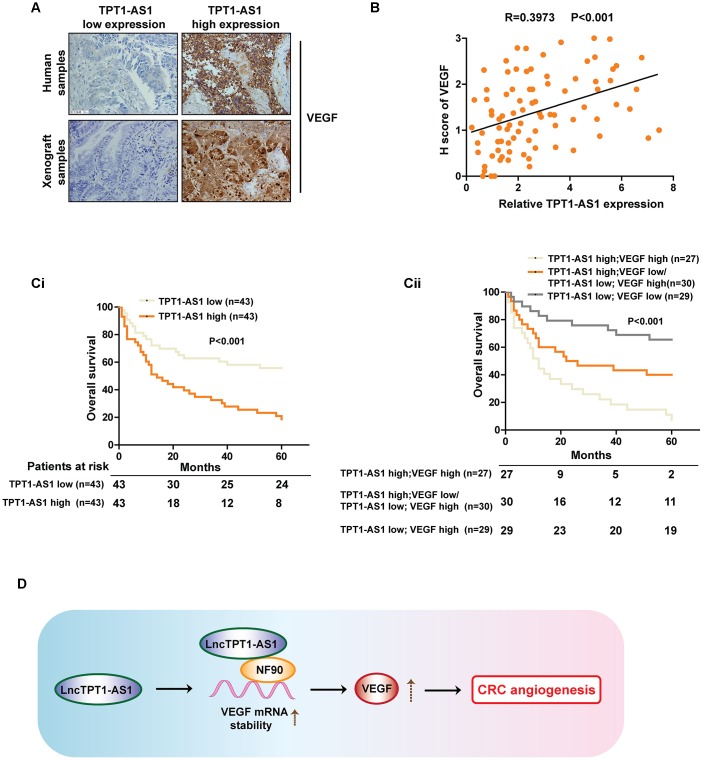
**Clinical relations between TPT1-AS1 and VEGFA in CRC patients’ tissues.** (**A**) IHC staining was used to detect the VEGFA expression in clinical and xenograft tumor samples. The clinical and xenograft samples were divided into TPT1-AS1 high or low groups by using qRT-PCR. (**B**) A positive relationship between TPT1-AS1 and VEGFA expression was confirmed in clinical samples by Pearson analysis. (**Ci**) The Kaplan-Meier analysis was used to show the overall survival of patients in TPT1-AS1 high/low groups. (**Cii**) The overall survival of patients in indicated TPT1-AS1/VEGFA expression groups.

To further validate our findings in CRC cell lines and mice models, IHC staining was performed to examine the protein level of VEGFA in clinical and xenograft tumor samples. As indicated in Figure 6A, tumor tissues with high expression of TPT1-AS1 tended to show high levels of VEGFA expression in both clinical samples and xenograft tumors, and vice versa. The above contrary, patients with TPT1-AS1 and VEGFA low expression showed observations were strengthened by Pearson analysis of the clinical samples (Figure 6B). The Kaplan-Meier analysis revealed that TPT1-AS1 high expression or VEGFA high expression in CRC significantly correlated with poor OS (Figure 6Ci and 6Cii). On the highest OS (Figure 6Cii). Together, our data highlighted a crucial role for TPT1-AS1/NF90/VEGFA signaling pathway in the angiogenesis of human CRC.

## DISCUSSION

In this study, we found that TPT1-AS1 is upregulated in CRC tissues and high-metastatic CRC cell lines. Meanwhile, the TPT1-AS1 expression is positively associated with the prognosis of CRC patients. Functional assays showed that TPT1-AS1 promoted CRC angiogenesis and liver-metastasis in mice model. In addition, VEGFA was identified as the TPT1-AS1 downstream target based on the following results: (1) silencing TPT1-AS1 in CRC cells inhibited the VEGFA secretion in the supernatant. (2) knockdown of TPT1-AS1 decreased the stability of VEGFA mRNA in CRC cells. (3) TPT1-AS1 interacted with NF90, which promoted the association between NF90 and VEGFA mRNA. Furthermore, we confirmed the expression pattern of TPT1-AS1 and VEGFA in clinical samples and xenograft tumors. These data reveal a pro-tumor network by a TPT1-AS1/NF90/VEGFA axis.

The nuclear factor 90 (NF90) is known as a RNA-binding protein (RBP), which can regulate the posttranscriptional fate of target mRNAs by binding to their untranslated or coding regions [11, 12]. The NF90 family is reported to be highly expressed in CRC tissues and shows interesting abilities to regulate the stability or the translation of VEGFA mRNA [13, 14]. However, upstream regulatory factors except NF90 remain to be studied. Our data demonstrated that the ability of NF90 regulating VEGFA mRNA stability is regulated by TPT1-AS1. The interaction between TPT1-AS1 and NF90 enhances the stability of VEGFA mRNA. Consistent with our results, previous studies have reported the similar regulatory mechanisms. For example, lncRNA GHET1 and lncRNA FGF13-AS1 were reported to interact with the downstream RNA-bind proteins and affect the c-Myc mRNA stability [15, 16]. Notably, NF90 regulates downstream targets through different ways: it could function as a DNA-binding protein and stimulate or suppress gene transcriptions [13, 17]; control the turnover of mRNA [10]; or control mRNA translation through predicted binding motifs [18]. In our study, we showed that TPT1-AS1 was not able to regulate VEGFA transcription, but the VEGFA mRNA stability. However, we did not detect the possibility that TPT1-AS1 controlled the translation of VEGFA mRNA. Thus, further research is still needed to unravel the mechanism that TPT1-AS1 regulates the VEGFA expression.

TPT1-AS1 was identified as one of the liver metastasis-associated lncRNAs in CRC by overlap analysis [7]. Nevertheless, whether TPT1-AS1 can be regarded as a valuable predictor for CRC metastasis remains to be studied. In this study, we determined the expression pattern of TPT1-AS1 in CRC tissues and cell lines for the first time. Consistent with the bioinformatic data, we found that TPT1-AS1 expression was upregulated in CRC tissues and highly metastatic cell lines. In vivo assays unraveled a pro-angiogenesis and metastasis function of TPT1-AS1 by enhancing VEGFA expression. However, in contrast to our findings, TPT1-AS1 was determined as a tumor suppressor in gliomas in a recent study. The authors identified that TPT1-AS1 inhibited glioma cell autophagy and proliferation by sponging miR-770-5p [19–21]. In accordance with our study, another report showed that TPT1-AS1 promoted tumorigenesis and metastasis of ovarian cancer by inducing TPT1 expression [22], which also identified TPT1-AS1 as an oncogenic factor. Similarly, another research indicated that TPT1-AS1 promoted cervical cancer growth and metastasis by sponging miR-324-5p [23]. Their findings again confirmed the tumor-promoting function of TPT1-AS1. Obviously, knowledge of the biological determinants that define the antitumorigenic role of TPT1-AS1 remains a challenge. Of note, not through transcriptional activation or sponging microRNAs, we demonstrated that TPT1-AS1 promoted CRC metastasis through enhancing VEGFA mRNA stability, which unraveled a new regulatory mechanism of TPT1-AS1. This finding will undoubtedly enrich the recognition of the network between TPT1-AS1 and downstream targets and verify the crucial roles of TPT1-AS1 in CRC.

In summary, we demonstrate that TPT1-AS1 is upregulated in CRC tissues, which is correlated to poor prognosis. Functional assays unravel a pro-angiogenesis and metastasis role of TPT1-AS1. Mechanistically, TPT1-AS1 interacts with NF90 and subsequently promotes the association between NF90 and VEGFA mRNA, which leads to the upregulation of VEGFA mRNA stability. Therefore, we elucidate a new regulatory mechanism of TPT1-AS1 in CRC angiogenesis and targeting the TPT1-AS1/NF90/VEGFA axis may provide a useful strategy for diagnosis and treatment for colorectal cancer patients.

## MATERIALS AND METHODS

### Cell lines and CRC specimens

The CRC cell lines (SW480 and HCT116) were obtained from the Department of Cell Biology, China Medical University. The cells were identified by the Cell Bank, Type Culture Collections, Chinese Academy of Sciences (CBTCCCAS). SW480 cells were cultured in DMEM high glucose medium with 10% fetal bovine serum. HCT116 cells were maintained in McCoy’s 5A medium with 10% FBS. All cells were cultured in a humidified incubator containing 5% CO2 at 37°C.

Fresh CRC tissues and the corresponding paired adjacent non-tumor tissues were obtained from 80 patients who underwent surgery in the Department of General Surgery of the First Affiliated Hospital of Harbin Medical University between Mar 2014 and Jan 2016. All the patients provided consent according to the ethical standards of Declaration of Helsinki. The study was supported by the ethics and scientific committee of Harbin Medical University. All the specimens were frozen at -80°C after surgical resection.

### Quantitative real-time polymerase chain reaction (qRT-PCR)

Total RNA was extracted with TRIzol reagent (Invitrogen, USA). One μg RNA was used to synthesize cDNA using QuantiTect Reverse Transcription Kit (Qiagen, Germany) following the manufacturer’s instructions. Quantitative PCR was performed using the SYBR Green kit (Thermo Fisher, USA) and a 7500 Real-Time PCR System (Applied Biosystems). The expression of each target mRNA was normalized to GAPDH. The primers used were as follows: TPT1-AS1, 5′-GCCACCACTCCCAGATCTTC-3′ (forward) and 5′-TGCTTGGGATTTACTGGAGGAC-3′ (reverse); VEGFA, 5′-AGGGCAGAATCATCACGAAGT-3′ (forward) and 5′-AGGGTCTCGATTGGATGGCA-3′(reverse); NF90, 5′-TTAGCTGGAGAAACGCTATCAGT-3’ (forward) and 5′-AATGACACAAGACTTCAGCCC-3’ (reverse); GAPDH, 5′-GGAGCGAGATCCCTCCAAAAT-3′ (forward) and 5′-GGCTGTTGTCATACTTCTCATGG-3′ (reverse). Relative RNA expression was calculated using 2^−ΔΔCT^ method.

### Immunohistochemistry (IHC) and *in situ* hybridization (ISH)

The paraffin-embedded tissues were deparaffinized in xylene and rehydrated through a series of graded dilutions of ethanol. For IHC, the slides were microwaved in 0.01M sodium citrate for 5 min for antigen retrieval. After incubation in 3% H_2_O_2_ for 5 min, the slides were blocked with bovine serum albumin (BSA) for 30 min. Then the slides were incubated with first antibodies overnight at 4 °C and subsequently with biotin-labeled secondary antibodies for 30 min, followed by a peroxidase-labeled avidin–biotin complex (Vector Laboratories, USA) for 30 min. The slides were developed in 3,3-diaminobenzidine tetrahydrochloride (DAB) and counterstained with hematoxylin. ISH was performed as previously described [14]. The DIG Nucleic Acid Detection kit (Roche, Germany) was used for the detection of TPT-AS1 following the manufacturer's instructions. The locked nucleic acid (LNA)-modified oligonucleotide probe targeting TPT1-AS1 was designed and synthesized by Exiqon (Vedbaek, Denmark). The slides were visualized and imaged under a microscope.

### Migration and invasion assays

Transwell assays were performed as previously described [16]. Briefly, for the migration assays, 10^5^ indicated cells were seeded into the upper chamber of each insert with serum free medium. Medium with FBS were added into the lower chambers. After 24h, the cells on the top of the chambers were removed by cotton swabs and the cells on the underside were fixed with 4% paraformaldehyde. Then the cells were stained with crystal violet and imaged under a microscope. For the invasion assays, the upper chambers were coated with Matrigel (BD Bioscience, USA). Then the cells were seeded on top of the Matrigel. All the experiments were performed triplicate.

### HUVEC tube formation assays

HUVECs were purchased from American Type Culture Collection and cultured in endothelial cell growth medium (Invitrogen). HUVEC cells (10^5^ cells/ml) were added to the Matrigel-precoated 96-well plates with indicated condition medium treatment. After 18h, the cells were observed and imaged under a microscope. The number of vessel branch points of tubes were counted for each field. The results were expressed as means ± S.D.

### In vivo study

All the procedures involved in animal experiments were performed in accordance with the Guide for the Administration of Affairs Concerning Experimental Animals, the national guideline for animal experiments. Male NOD/SCID mice (Vital Rivers, China) were housed in a specific pathogen free condition. For subcutaneous tumorigenesis assays, HCT116-Luc cells (5 × 10^5^) transfected with shTPT1-AS1 or sh-control were injected into mice. Tumor growth was monitored every week and the tumors were collected after 5 weeks. For the tumor metastasis model, HCT116-Luc cells (5 × 10^5^) transfected with shTPT1-AS1 or sh-control were injected into the spleen of mice. By the end of 6 weeks, after intraperitoneal injection of D-luciferin, all mice were imaged by the Xenogen IVIS Spectrum Imaging System (Caliper Life Sciences, USA) before being sacrificed. Primary tumor volume and the number of liver metastatic nodes were measured. For each tissue, HE or IHC staining was performed for histological detection. All the animal assays were approved by the Animal Experimental Ethics Committee of Harbin Medical University

### Magnetic Luminex® performance assay

HCT116 cells transfected with sh-control or shTPT1-AS1 were cultured in serum-free medium for 24h and the supernatant were collected after centrifugation. Then the supernatant was analyzed with the FlexMAP 3D (Luminex®) platform using a custom made MILLIPLEX MAP Human Cytokine/Chemokine Magnetic Bead Panel (Millipore, U.S.A) which includes the following human cytokines: SCF, IL-6, sCD31, MCP1, MIP1α, SDF-1, VEGFA, TNFα, MMP2, MMP3, MMP8, MMP9 and MMP12. The supernatant was processed in triplicate following the manufacturer’s instructions and analyzed with the MILLIPLEX-Analyst Software using a five-parameter nonlinear regression formula to calculate sample concentrations from the standard curves.

### Western blot

Total protein of indicated cells was extracted using RIPA lysis buffer (Solarbio, China) with protease inhibitors. Cell lysates were separated on 10% SDS gel electrophoresis and transferred to a PVDF membrane (Millipore, USA). The membrane was blocked with 5% non-fat dry milk in TBST and then incubated with primary antibodies: anti-NF90 (1:10000, Abcam, USA), anti-VEGFA (1:10000, Abcam) and anti-GAPDH (1:1000, Sigma) overnight at 4 °C. The membranes were subsequently incubated with peroxidase-conjugated secondary antibody (1:5000; Pierce Biotechnology) and detected with ECL detection reagents (Thermo Fisher Scientific).

### Immunofluorescence (IF) and fluorescence in situ hybridization (FISH)

The cells on the cover clips were fixed with 4 % cold paraformaldehyde. After two washes in PBS, the cell membranes were permeabilized with 0.1 % TritonX-100. For IF, the cells were washed with PBS and blocked with 5 % BSA for 1 h followed by incubation with anti-VEGFA (1:250, Abcam, USA) at 4 °C overnight and then incubation with secondary antibodies conjugated to fluorescein isothiocyanate (1:200, EarthOx, USA) at room temperature for 40 min. The nuclei were dyed with 40,6-diamidino-2-phenylindole (DAPI). For FISH, lncRNA FISH Probe Mix and Fluorescence In Situ Hybridization Kit (RIBO Bio, China) were used for detecting TPT1-AS1 expression in CRC cells following the manufacture's protocol. Photos were obtained through laser scanning microscopy (FV1000, Olympus, Japan).

### Luciferase reporter assay

Luciferase reporter assays were performed with the Dual Luciferase Assay System (Promega, USA) according to the manufacturer's protocol. Briefly, HCT116 cells were co-transfected with pcDNA 3.1-TPT1-AS1 or pcDNA 3.1-antisense-TPT1-AS1 constructs, 0.2 μg wild-type VEGFA promoter-luciferase reporter plasmid or 0.2 μg basic-luciferase reporter plasmid and 0.02 μg pRL-TK plasmid (Promega, USA) with Lipofectamine 3000 (Invitrogen, USA). After 48 hours, cells were collected and lysed. Twenty μl of each lysate were detected with a luminometer using the Dual Luciferase Assay System (Promega). For each experiment, the firefly luciferase activity was normalized to the activity of the Renilla luciferase used as an internal control.

### RNA immunoprecipitation (RIP)

As previously described [16], RIP assays were performed using the Magna RIP RNA-binding protein immunoprecipitation kit (Millipore, USA) following the manufacturer's instructions. Briefly, 2×10^7^cells were lysed in RIP Lysis buffer. The beads were incubated with indicated antibodies for 30min at room temperature to form the beads-antibody complex. Then the RIP lysates were added to the complex in RIP immunoprecipitation buffer. After extensive washing, the bead-bound immunoprecipitate was eluted with elution buffer at 55 °C. Phenol/chloroform/isoamyl alcohol was used to isolate protein associated RNAs from the eluted immunoprecipitate. Purified RNAs were then subjected to qRT-PCR or reverse transcription PCR. The primers specific to TPT1-AS1 immunoprecipitate sequence are shown as below: 5’-GTTTCACCATGTTGGCCAGG-3’ (forward) and 5’-GGATAGCCTCCTTGGAGC TG-3’ (reverse). The primers specific to VEGFA immunoprecipitate sequence are listed below: 5’-CTGTAGACACACCCACCCAC-3’ (forward) and 5’-CCTCCTCTTCCCTGTCAGGA-3’ (reverse).

### RNA pull-down assay

RNA pull-down assay was performed as previously described [16]. TPT1-AS1 and its antisense RNA were in vitro transcribed and biotin-labeled with Biotin RNA Labeling Mix (Roche). Next the biotin-labeled RNA oligomers (100 pmol) were incubated with protein lysates (2mg) from HCT-116 cells. One hour after the incubation, streptavidin agarose beads (Invitrogen) were added to the reaction mix to isolate the RNA-protein complex. After three washes at room temperature, the retrieved protein was subjected to normal western blot analysis.

### Statistics analysis

Quantitative data in this study were presented as mean ± standard deviation (SD) from at least three replicates. The data were analyzed either by the two-tailed unpaired Student's t-test (two groups) or one-way ANOVA (greater than two groups). Statistical analysis was performed by GraphPad Prism 5. Pearson correlation analysis was used to elucidate the correlation of two genes or proteins. R software package (version 3.0.0) were used. *P < 0.05 was thought as significant statistically. And **P < 0.01 was very significant while # marked no significance.

## Supplementary Material

Supplementary Figure 1
