# Supplementary material for: Long non-coding RNA TPT1-AS1 promotes angiogenesis and metastasis of colorectal cancer through TPT1-AS1/NF90/VEGFA signaling pathway
Source: Aging (Albany NY). 2020 Apr 4;12(7):6191–205. doi: 10.18632/aging.103016 (PMC7185097; doi:10.18632/aging.103016)
Supplement: Supplementary Figure 1 [file aging-12-103016-s001..pdf]

SUPPLEMENTARY FIGURE

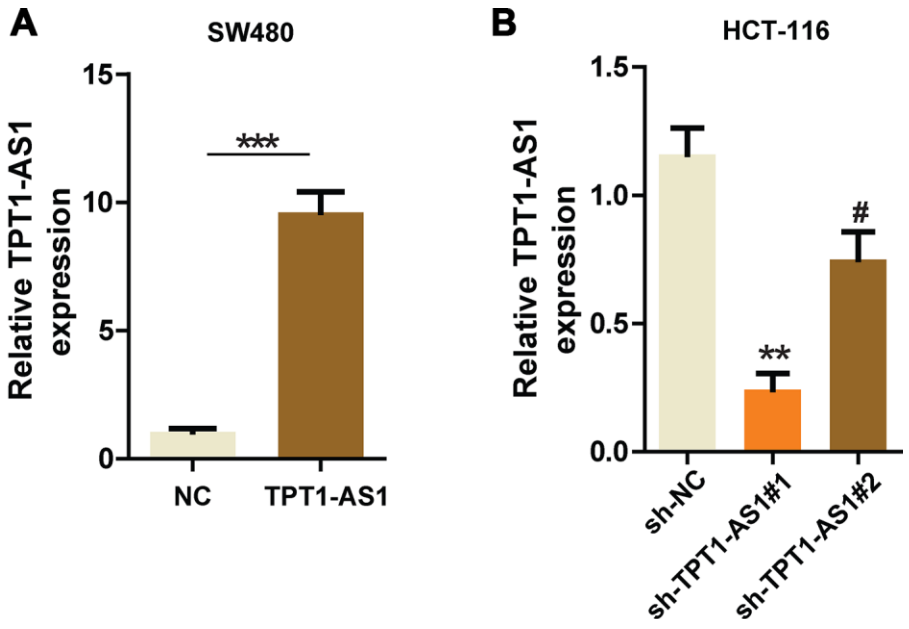

Supplementary Figure 1. The expression of TPT1-AS1 was detected in TPT1-AS1-overexpressed SW480 cells (**A**) or TPT1-AS1-knockdown HCT116 cells (**B**).
